# Anti-cooperative ligand binding and dimerisation in the glycopeptide antibiotic dalbavancin†
†Electronic supplementary information (ESI) available. See DOI: 10.1039/C3OB42428F



**DOI:** 10.1039/c3ob42428f

**Published:** 2014-03-10

**Authors:** Mu Cheng, Zyta M. Ziora, Karl A. Hansford, Mark A. Blaskovich, Mark S. Butler, Matthew A. Cooper

**Affiliations:** a Division of Chemistry and Structural Biology , Institute for Molecular Bioscience , The University of Queensland , Brisbane , Queensland 4072 , Australia . Email: m.cooper@uq.edu.au ; Tel: +61-7-3346-2044

## Abstract

Dalbavancin, a semi-synthetic glycopeptide with enhanced antibiotic activity compared to vancomycin and teicoplanin, binds to the C-terminal lysyl-d-alanyl-d-alanine subunit of Lipid II, inhibiting peptidoglycan biosynthesis. In this study, micro-calorimetry and electrospray ionization (ESI)-MS have been used to investigate the relationship between oligomerisation of dalbavancin and binding of a Lipid II peptide mimic, diacetyl-Lys-d-Ala-d-Ala (Ac_2_-Kaa). Dalbavancin dimerised strongly in an anti-cooperative manner with ligand-binding, as was the case for ristocetin A, but not for vancomycin and teicoplanin. Dalbavancin and ristocetin A both adopt an ‘closed’ conformation upon ligand binding, suggesting anti-cooperative dimerisation with ligand-binding may be a general feature of dalbavancin/ristocetin A-like glycopeptides. Understanding these effects may provide insight into design of novel dalbavancin derivatives with cooperative ligand-binding and dimerisation characteristics that could enhance antibiotic activity.

## Abstract 2

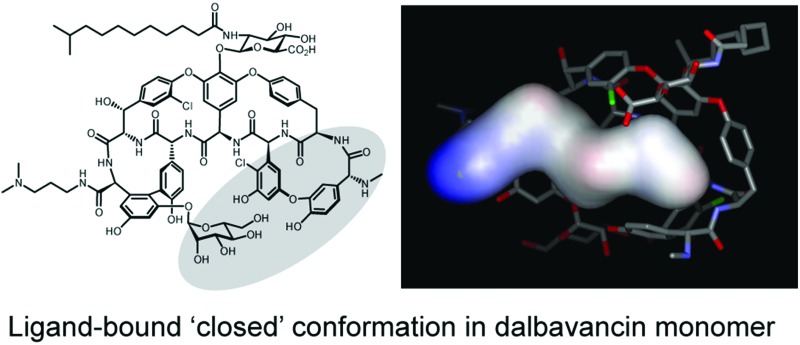
Dalbavancin, a semi-synthetic glycopeptide with enhanced antibiotic activity compared to vancomycin and teicoplanin, dimerises strongly in an anti-cooperative manner with ligand binding.

## Introduction

**Fig. 1:**
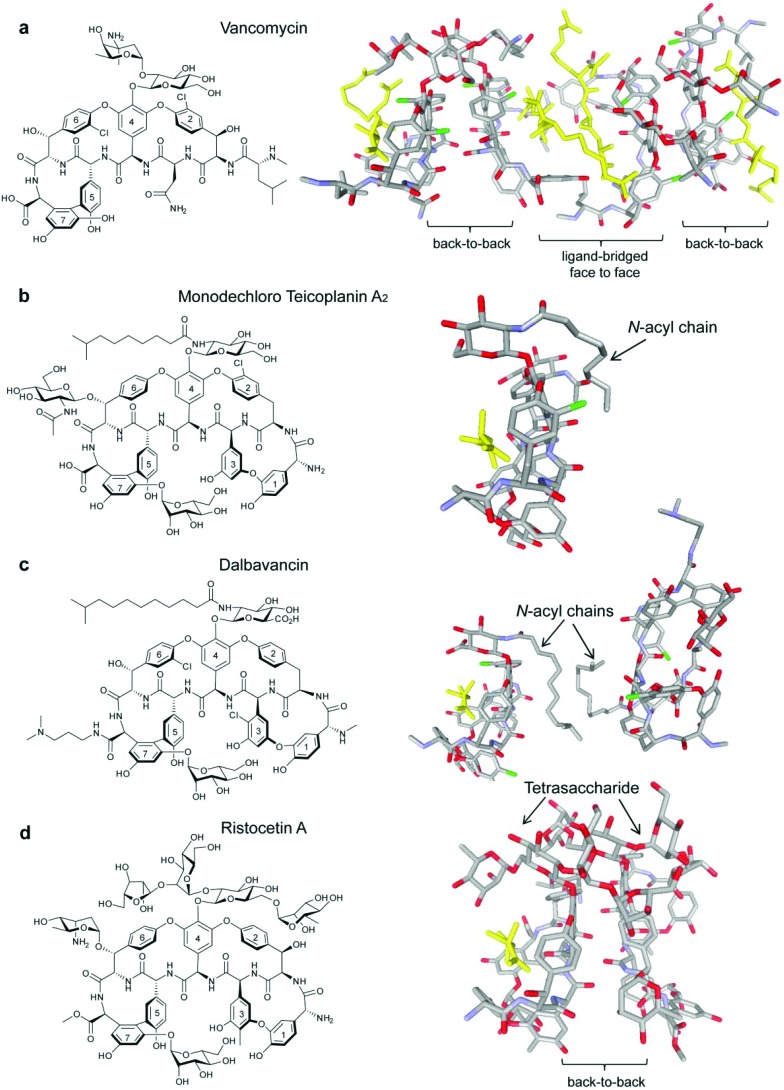
Glycopeptide antibiotics vancomycin (a),^19^ monodechloro teicoplanin A_2_ (b),^33^ dalbavancin (c)^19^ and ristocetin A (d)^19^ with published crystal structures highlighted to show bound ligand Lys-d-Ala-d-Ala (yellow) in the Lipid II binding site. Carrier proteins used for crystallisation omitted from (b), (c) and (d).

Acute bacterial skin and skin-structure infections (ABSSSIs), which are commonly caused by the Gram-positive cocci *Staphylococcus aureus, Streptococcus pyogenes* and to a lesser extent *Enterococcus faecalis* and *E. faecium*,^1,2^ lead to hospitalization and substantial health care costs.^3^ The emergence of antibiotic resistant strains, such as methicillin-resistant *S. aureus* (MRSA), has further complicated ABSSSI treatment.^4–8^ The glycopeptide/lipoglycopeptide antibiotics vancomycin (Fig. 1a) and teicoplanin (Fig. 1b) are normally used for MRSA treatment^9,10^ but MRSA strains with reduced susceptibility to glycopeptides have emerged and rapidly spread.^11,12^ In response, second-generation lipoglycopeptides have been developed that include telavancin (Fig. S1a, see ESI†), which was approved by the FDA in 2009, and dalbavancin (Fig. 1c) and oritavancin (Fig. S1b, see ESI†), which are both in late stage clinical development.^13^

All three second-generation lipoglycopeptides contain a common heptapeptide backbone that binds to the C-terminal l-lysyl-d-alanyl-d-alanine subunit of peptidoglycan precursors, resulting in inhibition of cell wall biosynthesis and cell death.^14^ The lipophilic side chains have been proposed to bind to serum proteins, as well as the bacterial membrane, thereby prolonging serum half-life and increasing activity against resistant strains.^15,16^ Recent NMR studies have suggested that the alkyl side chain of oritavancin interacts with pentaglycyl bridge segments of the cell wall peptidoglycan in *S. aureus* rather than the membrane.^17^ Telavancin and oritavancin are classified as vancomycin-type glycopeptides, while dalbavancin belongs to the teicoplanin-type class,^18^ with an additional macrocyclic ring formed between aryl residues 1 and 3.^19^ The vancomycin-type glycopeptides, including vancomycin,^20^ eremomycin,^21^ balhimycin^22^ and oritavancin,^23^ are able to dimerise in aqueous solution with dimerisation being cooperative with ligand-binding.^20,21^ Teicoplanin does not dimerise,^24^ but ristocetin A, another teicoplanin-type antibiotic, is the only glycopeptide previously reported to display dimerisation that is anti-cooperative with ligand-binding (Fig. 1d).^18,21,24^

Dalbavancin is a semi-synthetic *N*,*N*-dimethyl-1,3-diaminopropane derivative of the teicoplanin-like A40926 Factor B_0_.^25,26^ It displays enhanced *in vitro* activity compared to vancomycin and teicoplanin against methicillin-susceptible *S. aureus* (MSSA), MRSA, coagulase-negative staphylococci (CoNS) and non-VanA enterococci.^27,28^ To date, there have been only two studies published on the mode of action of dalbavancin: a recent patent has described oligomerisation detected by electrospray (ESI)-MS, protein-binding measured using MALDI-TOF and binding to diacetyl-Lys-d-Ala-d-Ala in the presence of serum protein using isothermal titration calorimetry (ITC),^28^ while an X-ray crystal structure of dalbavancin bound to a Lys-d-Ala-d-Ala (Kaa) binding epitope attached to a carrier protein was published in 2012.^19^ In this X-ray structure, two dalbavancin molecules were loosely associated in a back-to-back dimer *via* their fatty acyl chains (Fig. 1c).^19^

In this study, ITC and ESI-MS were used to investigate the relationship between dimerisation/oligomerisation of dalbavancin and binding of its target ligand diacetyl-Lys-d-Ala-d-Ala (Ac_2_-Kaa) in aqueous solutions and the results were compared to vancomycin, teicoplanin and ristocetin A. Serum components were not used in these experiments to reduce non-specific effects caused by protein binding.^29,30^ The ITC experiments showed that dalbavancin dimerised in an anti-cooperative manner with ligand-binding, which was also observed for ristocetin A. ESI-MS demonstrated similar oligomerisation behaviours between dalbavancin and ristocetin A in solution. Vancomycin also oligomerised, but weakly in the absence of ligand, whereas teicoplanin did not oligomerise. These data support the hypothesis that the anti-cooperativity between dimerisation and ligand-binding might be a feature of most teicoplanin-type glycopeptides, potentially due to a general ligand-induced ‘closed’ conformation observed in the crystal structures of dalbavancin and ristocetin A.^19^ Antibiotics with high dimerisation constants are generally potent against bacteria (*i.e.* eremomycin^31^ and oritavancin^15^), and the cooperativity between dimerisation and ligand-binding has previously been proposed to correlate with enhanced antibacterial activity.^32^ This study might provide some insights for further design and synthesis of novel glycopeptide/lipoglycopeptide derivatives with enhanced activity against resistant strains, in particular with VanA-type enterococci.

## Results and discussion

### Synthesis of dalbavancin

As dalbavancin was not available commercially, it was synthesised from the glycopeptide A40926-B_0_ using methods modified from a previous procedure.^34^ This synthetic dalbavancin gave minimum inhibitory concentration (MIC) values in agreement with previous studies (Table S1, see ESI†).^15^

### Dalbavancin dimerises strongly in solution

The presence of a fatty acyl chain contributes to dalbavancin's cLogP value of 1.54 and results in a lack of solubility in aqueous solution at physiological pH. Although serum protein can be used to increase the solubility of dalbavancin at neutral pH,^28^ protein binding dramatically reduces the free drug concentration of dalbavancin and could cause non-specific effects on dalbavancin–ligand interactions, complicating this analysis. Hence, a protein-free, buffered aqueous solution (0.1 M NaOAc, pH 5.0) was used to dissolve dalbavancin. LC/MS analyses showed that dalbavancin was completely soluble and stable in this buffer (Fig. S2, see ESI†), which was consistent with a previous study of dalbavancin solubility.^28^

**Fig. 2:**
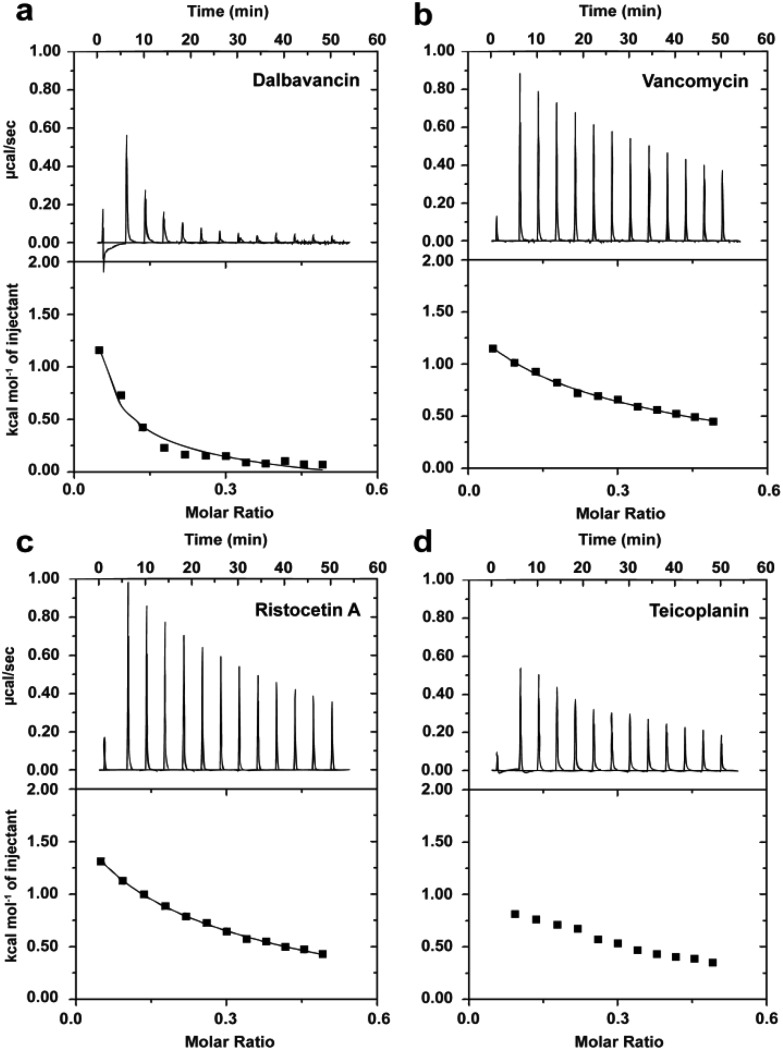
Typical ITC dilution data of dalbavancin (a), vancomycin (b), ristocetin A (c) and teicoplanin (d) in the absence of ligand at 25 °C in 0.1 M NaOAc buffer, pH 5.0. Upper profile: endothermic responses for sequential injections; lower profile: integrated dilution heat effects with theoretical fit to a dimer–monomer dissociation model.^20^

Dimerisation of dalbavancin was investigated by ITC dilution experiments, in which highly concentrated solutions of dalbavancin were titrated into a dilution buffer (0.1 M NaOAc, pH 5.0) to detect heat energy changes caused by dissociation of dalbavancin dimers.^20^ The resulting dissociation was endothermic (Fig. 2a), as was the case for vancomycin (Fig. 2b) and ristocetin A (Fig. 2c), though the heat pulses were broader and took longer to return to the baseline compared to vancomycin and ristocetin A. Teicoplanin showed negligible dose-dependent response beyond that expected for simple heat of dilution (Fig. 2d), which was consistent with a previous report that indicated that it exists exclusively as a monomer.^33^ Dimerisation constants (*K*_dim_) of vancomycin and ristocetin A in the absence of ligand were in low mM ranges (Table 1), in agreement with previous reports.^20,35–37^ In contrast, the *K*_dim_ value of dalbavancin was approximately 50-fold higher than that for vancomycin or ristocetin A, suggesting that dalbavancin dimerises strongly under these experimental conditions.

**Table 1:** Dimerisation thermodynamics of dalbavancin, vancomycin and ristocetin A in the absence or presence of ligand (Ac_2_-Kaa) at 25 °C in 0.1 M NaOAc, pH 5.0

Antibiotic	Ligand	(M^–1^)	(kJ mol^–1^)		
		*K*_dim_ ^*a*^	Δ*H*_dim_ ^*a*^	*T*Δ*S*_dim_ ^*a*^	Δ*G*_dim_ ^*a*^
Dalbavancin	None	38 400 ± 8260	–45.0 ± 2.2	–18.9 ± 1.7	–26.1 ± 0.5
	Ac_2_-Kaa	n/a^*b*^	n/a^*b*^	n/a^*b*^	n/a^*b*^

Vancomycin	None	750 ± 80	–11.5 ± 0.5	4.9 ± 0.7	–16.4 ± 0.3
	Ac_2_-Kaa	1940 ± 170	–17.1 ± 0.4	1.6 ± 0.2	–18.7 ± 0.2

Ristocetin A	None	920 ± 120	–14.2 ± 0.5	2.7 ± 0.8	–16.9 ± 0.3
	Ac_2_-Kaa	690 ± 140	–20.0 ± 1.3	–3.9 ± 1.8	–16.1 ± 0.5

^*a*^Data are means ± SD for *n* = 3.

^*b*^The thermodynamics of dalbavancin dimerisation in the presence of Ac_2_-Kaa could not be determined due to the poor solubility of the complex.

The dimerisation of teicoplanin is sterically impaired by both the *N*-acetyl-β-d-glucosamine substituent on residue 6 and the fatty acyl chain attached to the glucosamine on residue 4 which lies on the back (convex) interface (Fig. 1b).^33^ In comparison, the fatty acyl chain in dalbavancin is slightly longer and highly flexible, but it lacks the residue 6 *N*-acetyl-β-d-glucosamine subunit. In both antibiotics, the lipophilic chains are similarly oriented in the solid state, and there is evidence of self-association in dalbavancin molecules (Fig. 1c) but not in teicoplanin.^19^ These differentiating features apparently favour dimerisation in dalbavancin but not in teicoplanin.

The reported MIC values of dalbavancin are lower than that of vancomycin and teicoplanin, with MIC values against MRSA of 0.12 to 0.25 μg mL^–1^, compared to MIC values of vancomycin and teicoplanin against MRSA ranging from 1 to 2 μg mL^–1^ and from 2 to 8 μg mL^–1^, respectively.^27^ The improved antibacterial activity of dalbavancin may be correlated not only with bacterial membrane anchoring,^19^ which could serve to enhance the local concentration of antibiotic at the site of peptidoglycan biosynthesis on the membrane, but also with its strong dimerisation behaviour, as is the case for oritavancin.^23^

The ITC dilution results of this study indicated that dimerisation was driven by favourable enthalpy (Δ*H*_dim_) in all antibiotics with the exception of teicoplanin, but there were significant differences in the entropic component (*T*Δ*S*_dim_) (Table 1). The thermodynamic parameters of ristocetin A were similar to vancomycin in the absence of ligand, consistent with a previous ITC study.^20^ While dalbavancin dimerisation was more exothermic than vancomycin or ristocetin A, there was concomitant unfavourable dimerisation entropy. It is conceivable that self-association of the fatty acyl chains in the dalbavancin could bury the hydrophobic surface area from solvent, leading to a considerable entropy of solvation. In contrast, the exothermic dimerisation of dalbavancin may be attributed to the formation of amide-amide hydrogen-bonds between heptapeptide backbones, the favourable van der Waals interactions between non-polar groups and the orthogonal π–σ interactions between aromatic rings of dalbavancin complexes, as was the case for vancomycin and ristocetin A.^35,38^ Additional ionic interactions may also favourably contribute to the overall dimerisation enthalpy of dalbavancin.^28^ However, the hydrophobic interactions between the carbohydrate group and aromatic rings are important in stabilizing the dimer species of vancomycin^39^ and ristocetin A.^18^

### Dalbavancin dimerisation and ligand binding are anti-cooperative

Dalbavancin dimerisation was next studied in the presence of the ligand Ac_2_-Kaa by ITC dilution measurements using conditions that predominantly favoured the dalbavancin–ligand complex. This avoided complications caused by the change in ligand-bound state during the dilution/dissociation process as previously described.^20^ Vancomycin and ristocetin A were used for comparative purposes. Our data (Table 1) confirmed the cooperativity and the anti-cooperativity in vancomycin and ristocetin A, respectively, which was consistent with previous studies.^20,21^ However, it was impossible to determine the dimerisation of dalbavancin in the presence of ligand in solution due to the poor solubility of the dalbavancin–ligand complex. Although dalbavancin and the peptide ligand were both soluble in acetate buffer, when concentrated aqueous solutions of the two were mixed, precipitation was observed.

**Fig. 3:**
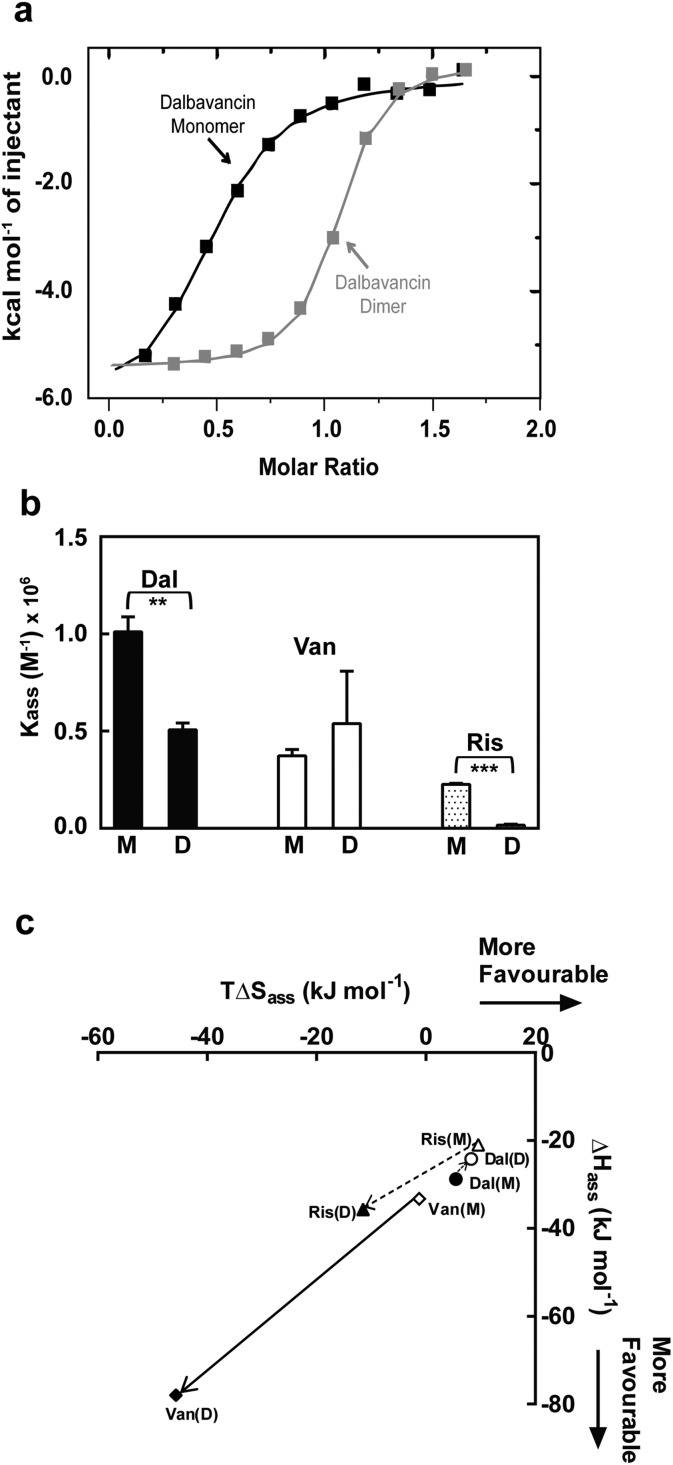
ITC binding data of dalbavancin (Dal), vancomycin (Van) and ristocetin A (Ris) at 25 °C in 0.1 M NaOAc (pH 5.0) showing anti-cooperativity of ligand binding to dimer for dalbavancin and ristocetin A. (a) Integrated titration curves upon complexation of Ac_2_-Kaa with dalbavancin monomer (black) and dimer (grey), with theoretical fit to a single site binding mode.^40^ (b) The binding constant (*K*_ass_) of antibiotic monomers (M) and dimers (D). Statistical comparison of *K*_ass_ values was performed by the two-tailed Student's *t*-test ( **, *p* < 0.01; ***, *p* < 0.001). (c) Enthalpy–entropy plots showing thermodynamics of ligand binding for three antibiotics (Dal: ○; Van: ; Ris: Δ). Means ± SD for *n* = 3.

The ligand-binding of dalbavancin in solution was investigated by ITC binding measurements at concentrations that populate monomeric or dimeric forms. The ITC data indicated that both were exothermic processes (Fig. 3a). The association constant (*K*_ass_) of the tripeptide ligand Ac_2_-Kaa toward the dalbavancin monomer was increased approximately 3-fold compared to monomeric vancomycin and 4-fold compared to monomeric ristocetin A (Fig. 3b). This *K*_ass_ value was reduced around 2-fold when binding to dalbavancin dimer, as was the case with ristocetin A, in which the *K*_ass_ value of ligand-binding of dimeric ristocetin A was reduced 10-fold. In contrast, the *K*_ass_ value of ligand-binding toward the vancomycin dimer was increased approximately 1.4-fold compared to its monomer, consistent with a previously reported value.^21^ The ITC binding data in this study demonstrated that dimerisation reduced ligand-binding affinity in cases of dalbavancin and ristocetin A, whereas dimerisation of vancomycin enhanced ligand-binding. Therefore, dalbavancin dimerises in an anti-cooperative manner with ligand-binding, in a similar fashion to ristocetin A.

Recent crystal structures of dalbavancin and ristocetin A with bound ligand, when compared to similar glycopeptide ligand-free structures, show that ligand binding induces a conformational change in which the two ends of the heptapeptide are drawn closer together, with the mannose attached to residue 7 reaching across to the biaryl ether linkage of residues 1 and 3.^18,19^ This ligand-bound monomer ‘closed’ conformation may interfere with dimerisation of these antibiotics, possibly disrupting formation of the ‘back-to-back’ network of hydrogen bonds. In contrast, the lack of crosslinking between residues 1 and 3 in vancomycin-type glycopeptides^16^ (*i.e.* vancomycin, eremomycin, balhimycin and oritavancin) presumably imparts greater flexibility and thus allows ligand-induced dimerisation.^21,23^ Thus, variations in conformational flexibility appears to dictate the cooperativity observed in vancomycin-type antibiotics and the anti-cooperativity in ristocetin A and dalbavancin.

Enthalpy (Δ*H*_ass_) *versus* entropy (*T*Δ*S*_ass_) plots of ligand-binding for dalbavancin, vancomycin and ristocetin A are shown in Fig. 3c. Δ*H*_ass_ against *T*Δ*S*_ass_ for vancomycin–ligand binding was close to a linear correlation, in which the *T*Δ*S*_ass_ reduction was similar to the Δ*H*_ass_ increment going from a monomer to a dimer. However, this was not the case for ristocetin–ligand binding due to a slightly reduced entropic contribution going from monomeric to dimeric forms. Ligand-binding of the dalbavancin dimer was more favoured entropically, but less favoured enthalpically than with its monomer. While dimerisation of both ristocetin A and dalbavancin was anti-cooperative with ligand-binding, the thermodynamic contributions to this behaviour differed. The two halves of the ristocetin A dimer are known to bind ligand with different affinities due to the asymmetric tetrasaccharide orientation in the ristocetin A dimer,^41,42^ which contributes to the anti-cooperative ligand-binding of ristocetin A.^18^ Removing the tetrasaccharide moiety and the residue 7 mannose in ristocetin A (known as ristocetin ψ) has been reported to change the anti-cooperative behaviour to cooperative.^21^ Dalbavancin lacks this tetrasaccharide group and thus the ligand-induced ‘closed’ conformation might provide the major contribution to the anti-cooperativity in dalbavancin.

In the absence of ligands the dimerisation constant of ristocetin A is similar to vancomycin (Table 1), but it is less active than vancomycin *in vitro*,^43^ most likely due to its anti-cooperativity. Additionally, a previous study reported that linking the vancosamine groups of two vancomycin molecules reduced the MIC value against vancomycin-resistant *E. faecium* from >16 μg mL^–1^ to 1 μg mL^–1^.^32^ It is notable that dalbavancin has poor activity against VanA-type enterococci with a MIC value of 32 μg mL^–1^,^44,45^ whereas oritavancin, which shares a similar dimerisation constant with dalbavancin, is highly active with a MIC of 0.25 μg mL^–1^.^23^ Hence, it could be hypothesised that the anti-cooperativity between dimerisation and ligand-binding might contribute to the poor activity of dalbavancin against VanA-type enterococci.

### Binding stoichiometry of dalbavancin monomers and dimers are different

The stoichiometry (*N*) obtained from ITC binding experiments reflects the number of moles of ligand Ac_2_-Kaa required to saturate all the available binding sites of the antibiotic.^28^ The binding stoichiometry values of vancomycin and ristocetin A ranged from 0.9 to 1.2 for both monomeric and dimeric antibiotic species (Fig. S3a–S3d, see ESI†), which corresponded to a 1 : 1 binding complex for the monomer and 2 : 2 for the dimer.^46^ Our data is in agreement with a previous study.^40^ For teicoplanin and dalbavancin, the calorimeter cell was pre-rinsed with the experimental concentration of glycopeptides to prevent non-specific binding of these lipophilic antibiotics to the metal surface of the calorimeter cell. The binding stoichiometry of the teicoplanin monomer was 0.8, which fitted to a 1 : 1 binding.^46^ Interestingly, the ligand-binding of the dalbavancin monomer fitted to a 2 : 1 dalbavancin : ligand complex (*N* closer to 0.5), while its dimer bound to ligand in a 1 : 1 ratio (*N* closer to 1, Fig. S3e–S3f, see ESI†). In the presence of serum protein, dalbavancin monomers have been reported to bind in a 1 : 1 ratio to the same tripeptide ligand Ac_2_-Kaa.^28^ The fatty acyl chain in the dalbavancin monomer is highly flexible,^19,33^ and can interfere with ligand binding. Immobilization of fatty acyl chains either by dimerisation or by protein binding is likely to prevent lipophilic groups from blocking the binding pocket of dalbavancin, thereby allowing for complete occupancy of the binding sites of dalbavancin.

### Dalbavancin and ristocetin A oligomerise in the absence of ligands

**Fig. 4:**
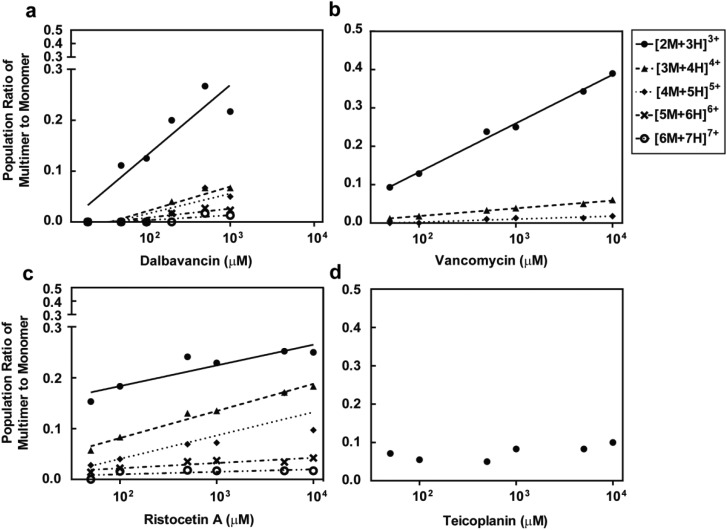
Concentration of dalbavancin (a), vancomycin (b), ristocetin A (c) and teicoplanin (d) *versus* the ratio of antibiotic multimer to monomer population. There are concentration-dependent increases in the ratios of multimer to monomer in all antibiotics except teicoplanin.

The potential formation of dalbavancin multimers (dimers or oligomers) in solution of varying concentrations was investigated using ESI-MS and compared to vancomycin, ristocetin A and teicoplanin. Potential multimers would be observed as the [*nM* + (*n* + 1)H]^(*n*+1)+^ mass ion species, where *n* is a positive integer indicating the multiplicity of the multimer (*e.g. n* = 2 when the multimer is a dimer and *n* = 4 when the multimer is a tetramer), *M* is the mass of the monomer and (*n* + 1)^+^ indicates the charge.^28^ For example, the dimer and tetramer species were assigned respectively as the [2M + 3H]^3+^ and [4M + 5H]^5+^ mass ion peaks. The MS data (Fig. S4–S7, see ESI†) showed the presence of multimers for dalbavancin, ristocetin A and vancomycin, but not for teicoplanin. Although the primary multimeric form was a dimer, the formation of various oligomers including trimers and tetramers were also observed. Dalbavancin and ristocetin A were able to form higher order oligomers in solution, such as pentamers and hexamers, at concentrations above 100 μM (Fig. 4), which were absent for vancomycin. The weak oligomerisation of vancomycin observed in this ESI-MS was consistent with a previous study, which demonstrated that the formation of vancomycin oligomeric species in solution was ligand mediated.^47^ In this ligand-free ESI-MS study, dalbavancin was found to oligomerise in a dose-dependent manner, as was the case with ristocetin A (Fig. 4). An increase in antibiotic concentration was found to correspond to the increased multimer mass traces and hence an increase in the population ratio of antibiotic multimer to monomer.

## Experimental section

### Antibiotics, ligand and bacteria

Vancomycin hydrochloride hydrate salt, teicoplanin containing >80% teicoplanin A_2_, ristomycin monosulfate containing >90% ristocetin A and A40926 ≥ 80% purity (HPLC) were purchased from Sigma-Aldrich and used without further purification. Dalbavancin (TFA salt) with 97% purity (HPLC) was synthesized based on a published procedure^34^ as described in the ESI.† The peptide ligand diacetyl-l-Lys-d-Ala-d-Ala (Ac_2_-Kaa) was purchased from Chiron-Mimotopes (Melbourne, Australia). MRSA ATCC 43300 and *Streptococcus pneumoniae* (multi-drug-resistant) ATCC 700677 were purchased from American Type Culture Collection (Manassas, VA, USA). *E. faecium* (VanA) was a clinical isolate supplied by Prof. D. Paterson from the University of Queensland Centre for Clinical Research (UQCCR, Brisbane, Australia).

### ITC

Calorimetry experiments were performed using a MicroCal Omega Auto-Itc200 (GE Healthcare, Australia) at 25 °C in triplicate. Each experiment consisted of sequential injections from the titration syringe into the calorimeter cell (cell volume ∼0.2 mL) with stirring at 1000× rpm. The interval time between each injection was 240 s. Computer simulations (curve fitting) were performed using the MicroCal Origin version 7.0 software package adapted for ITC data analysis to yield stoichiometry (*N*), enthalpy (Δ*H*_ass_), entropy (Δ*S*_ass_) and association constant (*K*_ass_) for the single site binding mode or dissociation constant (*K*_diss_) and enthalpy (Δ*H*_diss_) for the dimer–monomer dissociation mode. The Gibbs free energy (Δ*G*) was calculated using the Gibbs–Helmholtz thermodynamic and van't Hoff equations as previously described.^46^

### ITC: dimer–monomer dissociation

Calorimetric data for the dilution of dalbavancin and control antibiotics (vancomycin, ristocetin A and teicoplanin) solutions in the absence and presence of ligand Ac_2_-Kaa were determined using a modified calorimetric dilution method.^20^ Briefly, a 3 mM antibiotic solution in Buffer A (0.1 M NaOAc, pH 5.0) was consecutively injected (13 × 3 μL per injection, first injection of 0.5 μL) into the calorimeter cell initially containing Buffer A alone. Dilution experiments involving ligand employed consecutive injections of the antibiotic–ligand mixture (3 mM of antibiotic and 9 mM of free Ac_2_-Kaa) dissolved in Buffer A into the stirred calorimeter cell initially containing the same concentration of ligand Ac_2_-Kaa. Data were corrected for small injection/mixing effects from buffer controls that were performed separately and analyzed under identical conditions by omitting the first injection.

### ITC: single site binding

Ligand-binding experiments of monomeric and dimeric antibiotics were performed using a modified calorimetric titration method.^40^ Briefly, the concentrated ligand Ac_2_-Kaa solution (14- to 20-fold higher than the antibiotic solution) dissolved in Buffer A was sequentially injected (12 × 2 μL per injection, first injection of 0.5 μL) into the calorimeter cell charged either with antibiotic monomer solutions (0.01 mM for dalbavancin and 0.025 mM for vancomycin, ristocetin A and teicoplanin) or with antibiotic dimer solutions (0.2 mM for dalbavancin and 2 mM for vancomycin and ristocetin A) dissolved in Buffer A. Pre-rinsing the calorimeter cell with dalbavancin and teicoplanin solutions at the experimental concentration was required to avoid non-specific binding of antibiotics to the metal surface of the calorimeter cell (conditions were determined in separate experiments). Heat of reaction was corrected by the heat of dilution of ligand solution determined in separate experiments.

### ESI-MS

Experiments were performed using an Applied Biosystem API QStar Pulsar Mass Spectrometer equipped with a TurboIonSpray source, a Triple Quadrupole analyzer, operating in positive ion mode. The MS conditions were performed as previously described^28^ with some modifications. Dalbavancin was dissolved in H_2_O–isopropanol (v/v = 8/2) and serially diluted to give concentrations from 1 mM to 0.02 mM. Vancomycin, ristocetin A and teicoplanin were used as controls using concentrations from 10 mM to 0.05 mM in H_2_O–isopropanol (v/v = 8/2). The antibiotic solutions were injected (10 μL) into a flow of H_2_O–isopropanol (v/v = 8/2) (15 μL mL^–1^) and the MS data acquired from 500 to 2000 Da. Data were analyzed with the software version Analyst QS 1.1.

## Conclusions

In summary, our ITC and ESI-MS data show that dalbavancin dimerises in an anti-cooperative manner with ligand binding, which had only been previously reported for ristocetin A.^18,21,24^ Analysis of published crystallographic structures suggested that dalbavancin and ristocetin A share a similar constrained ligand-bound induced ‘closed’ conformation, which is absent in vancomycin-type glycopeptide antibiotics that dimerise and bind ligand cooperatively. Hence, it is conceivable that antibiotics with a similar ligand-induced ‘closed’ conformation might display dalbavancin/ristocetin A-like anti-cooperativity in dimerisation and ligand-binding. Given the reported correlation between vancomycin-type cooperativity and improved *in vitro* antibacterial activity, our findings suggest that further modifications to dalbavancin derivatives, such as removing the cross-linkage between aromatic rings 1 and 3 in dalbavancin, may help to overcome resistant bacteria, in particular VanA-type enterococci.
