# Supplementary material for: Anti-cooperative ligand binding and dimerisation in the glycopeptide antibiotic dalbavancin
Source: Org Biomol Chem. 2014 Mar 10;12(16):2568–75. doi: 10.1039/c3ob42428f (PMC4082399; doi:10.1039/c3ob42428f)
Supplement: Supplementary file 1 [file OB-012-C3OB42428F-s001.pdf]

This journal is © The Royal Society of Chemistry 2014

Electronic supplementary information

Anti-cooperative ligand binding and dimerisation in the glycopeptide antibiotic dalbavancin

Mu Cheng, Zyta M. Ziora, Karl A. Hansford, Mark A. Blaskovich, Mark S. Butler and Matthew A. Cooper*

Division of Chemistry and Structural Biology, Institute for Molecular Bioscience, The University of Queensland, Brisbane, Queensland, 4072, Australia. E-mail: m.cooper@uq.edu.au; Tel: +61-7-3346-2044

Table of Contents

Page 2 **Fig. S1** Structures of vancomycin-type glycopeptide antibiotics telavancin (a) and oritavancin (b)

Page 3 **Scheme S1** Synthesis of dalbavancin TFA salt **4** from A40926

Page 3-5 Experimental details for the synthesis of dalbavancin and clogP determination

Page 6 Antibacterial activity determination and **Table S1** *In vitro* activity of dalbavancin from literature and this study

Page 7 LCMS quantification of dalbavancin in 0.1 M NaOAc (pH 5.0) and **Fig. S2** Standard curve of dalbavancin in TFA/H_2O (1:20, v/v).

Page 8 **Fig. S3** Exothermic responses and titration curves with theoretical fit to a single site binding model upon interaction of antibiotics with ligand Ac_2-Kaa in 0.1 M NaOAc (pH 5.0) at 25°C.

Page 9 **Fig. S4** ESI-MS spectrum of dalbavancin (1 mM) in H_2O-isopropanol (v/v = 8/2).

Page 10 **Fig. S5** ESI-MS spectrum of vancomycin (10 mM) in H_2O-isopropanol (v/v = 8/2).

Page 11 **Fig. S6** ESI-MS spectrum of ristocetin A (10 mM) in H_2O-isopropanol (v/v = 8/2).

Page 12 **Fig. S7** ESI-MS spectrum of teicoplanin complex (A_2 major component) (10 mM) in H_2O-isopropanol (v/v = 8/2).

Page 13 Supplementary information references

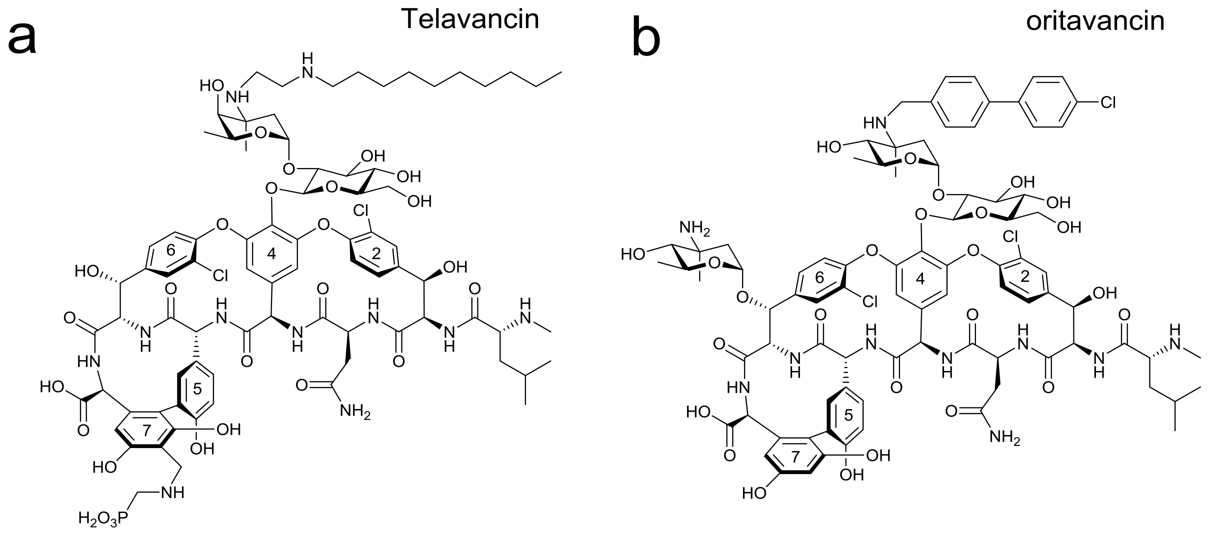

Fig. S1 Structures of vancomycin-type glycopeptide antibiotics telavancin (a) and oritavancin (b)

Synthesis of dalbavancin

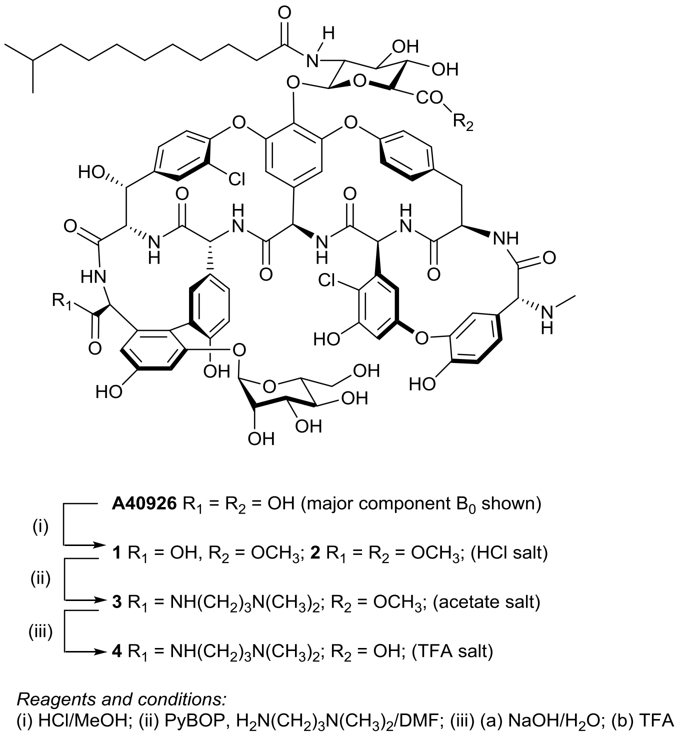

Scheme S1 Synthesis of dalbavancin TFA salt **4** from A40926.

General experimental

Routine analytical LCMS analysis was performed using a Shimadzu Prominence system utilising a SPD-M20A diode array UV-Vis detector, ELSD–LT II evaporative light scattering detector and LCMS–2020 mass spectrometer. Preparative HPLC was performed on an Agilent 1206 Infinity system, with monitoring at 210 nm. Final HPLC purity of dalbavancin **4** was assessed on an Agilent 1200 system, with monitoring at 210 nm. The following reverse phase columns were used throughout: column A: Agilent Zorbax Eclipse XDB-phenyl (3.0 × 100 mm, 3.5 µm); column B: Agilent Zorbax Eclipse XDB-Phenyl (21.2 × 100 mm, 5 µm); column C: Waters Atlantis T3 (2.1 × 50 mm, 5 µm); column D: Agilent Zorbax Eclipse XDB-phenyl (4.6 × 150 mm, 5 µm). The following HPLC solvents were used during analysis and purification: solvent A = H_2O + 0.05% HCO_2H; solvent B = CH_3CN + 0.05% HCO_2H; solvent C = H_2O + 0.1% TFA; solvent D = CH_3CN + 0.1% TFA; solvent E = H_2O + 0.1% CH_3CO_2H; solvent F = CH_3CN + 0.1% AcOH. **Method A:** column A, flow 1 mL/min, gradient timetable: 0 min, 70A:30B; 5 min, 60A:40B. **Method B:** column A, flow 1 mL/min, gradient timetable: 0 min, 80A:20B; 5 min, 50A:50B. **Method C:** column B, flow 20 mL/min, gradient timetable: 0 min, 95E:5F; 2 min, 95E:5F; 3 min, 80E:20F; 12 min,

64E:36F. **Method D:** column B, flow 20 mL/min, gradient timetable: 0 min, 95C:5D; 14 min, 28.5C:71.5D. **Method E:** column C, flow 1 mL/min, gradient timetable: 0 min, 75C:25D; 25 min, 25C:75D. **Method F:** column D, flow 1 mL/min, gradient timetable: 0 min, 95A:5B; 1 min, 95A:5B; 9 min, 100B.

A40926-B_0 monomethyl ester (1)

A40926 (0.226 g) was added in one portion to a solution of CH_3COCl (5 mL) in anhydrous MeOH (50 mL) at 0 °C. After 2 h at 4 °C, LCMS analysis (method A) confirmed complete consumption of starting material. The product was precipitated by dropwise addition of the reaction mixture to diethyl ether (250 mL) at 0 °C. The resultant suspension was centrifuged, the supernatant decanted, and the pellet re-suspended in diethyl ether and centrifuged once more. The supernatant was discarded, and the pellet was immediately dissolved in a minimum volume of CH_3CN/H_2O (1:1) and freeze-dried, affording **1** as the HCl salt (0.198 g), purity 78% at 200 nm. Failure to carry out the immediate freeze-drying step leads to inadvertant exposure of **1** to traces of acid upon storage, causing degradative cleavage of the *N*-acylamino methylglucuronate. HPLC (method A): t_R = 2.5 min (A40926), 2.74 min **1**, 3.26 min **2**. The material was used in the next step without further purification.

Amide (3)

Crude **1** (0.207 g) was dissolved in DMF (1.73 mL). PyBOP (0.0538 g) was added and the mixture agitated until homogeneous. The solution was cooled in an ice bath, and *N,N*-dimethyl-1,3-diaminopropane (0.029 mL) was added. The mixture was brought to room temperature. After 2 h, EtOAc (8 mL) was added and the resulting suspension was centrifuged. The supernatant was decanted, and the pellet was re-suspended in diethyl ether, and centrifuged once more. The supernatant was discarded, and the pellet freeze-dried from a minimum volume of 0.1% HOAc in CH_3CN/H_2O (1:1), affording crude product (0.29 g). HPLC (method B): t_R = 3.1 min. Purification by HPLC (method C) gave semi-pure product **3** as the acetate salt (0.109 g). The material was used in the next step without further purification. Note: The amide coupling step is highly prone to side reactions, with the overall reaction profile being sensitive to excess quantities of PyBOP and 1,3-diaminopropane; cautious monitoring of reagent stoichiometry is recommended.

Dalbavancin (4)

3 (0.109 g) was dissolved in H_2O (1.78 mL) and cooled in an ice bath. 1N NaOH solution (0.445 mL) was added, and the mixture warmed to room temperature. After 4 h, the reaction

mixture was cooled to 0 °C and acidified to pH 4.0–4.5 with a solution of TFA in H_2O (0.235 M, 2.1 mL). The mixture was immediately freeze-dried, affording crude **4** as a white solid (0.175 g). Crude **4** was dissolved in CH_3CN/H_2O (1:1, *ca.* 2.5 mL) and purified by HPLC (25 injections of 0.1 mL each, method D): t_R = 8.2 min, final yield 0.079 g (TFA salt). Final HPLC purity (method E): t_R = 12.7 min, 97% at 210 nm. HRMS (ESI+) *m/z* found $[M + 2H]^{2+}$ 908.3073, $C_{88}H_{102}Cl_2N_{10}O_{28}^{2+}$ requires 908.3116.

clogP calculation

The property of clogP as previously described[1] was calculated by Pipeline Pilot (Accelrys, Dan Diego CA, USA).

Antibacterial activity determination

MICs of dalbavancin against MRSA ATCC 43300, *S. pneumoniae* (multi-drug-resistant) ATCC 700677 and *E. faecium* (VanA) clinical isolate were determined by broth microdilution as described by the Clinical and Laboratory Standards Institutes (CLSI) M7-A7 methodology.[2] Briefly, serial two-fold dilutions of dalbavancin solution were added into Costar non-treated polystyrene 96-well plates (In Vitro Technologies, Australia), and each well inoculated with 0.1 mL of bacteria in Mueller-Hinton Broth with a final concentration of *ca.* 5×10^5 CFU/mL. The MIC was the lowest antibiotic concentration that showed no visible growth after 24 h of incubation at 37 °C.

Table S1 *In vitro* activity of dalbavancin from literature and this study

Bacteria	Dalbavancin MIC (µg/mL)	
	Literature[a]	Our study[b]
MRSA	≤ 0.008 – 0.5	0.25
S. pneumoniae (MDR)	≤ 0.008 – 0.25	0.25
E. faecium (VanA)	0.03 – > 32	> 8

[a] Ref[3]

[b] Mean value (n=2).

LCMS quantification of dalbavancin in 0.1 M NaOAc (pH 5.0)

Dalbavancin TFA salt was dissolved in TFA-H_2O (v/v = 1/20) to make a series of standard curve ranging from 100 to 6.25 μM (2-fold dilution). The standard curve was calculated according to the area under the curve at 210 nm. Dalbavancin was dissolved in 0.1 M NaOAc buffer (pH 5.0) to make a stock solution at a concentration of 3.0 mM. After sonication for 1 h, the stock solution was diluted 120-fold by the same buffer and the concentration was measured by LCMS and calculated according to the standard curve. LC condition: method F (monitoring at 210 nm). Analyses were performed in triplicate.

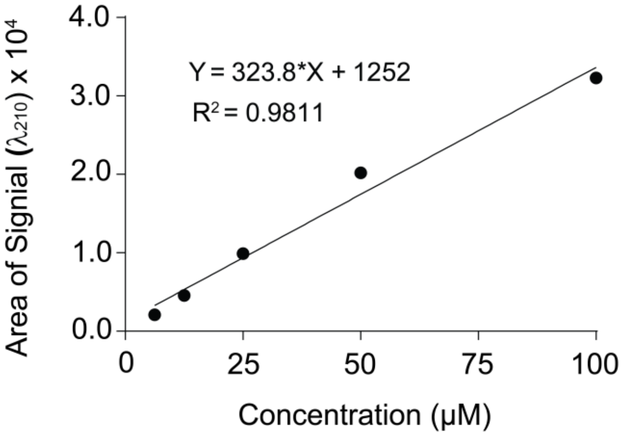

Fig. S2 Standard curve of dalbavancin in TFA-H_2O (v/v = 1/20). The 120-fold diluted dalbavancin from 3.0 mM stock solution in 0.1 M NaOAc (pH 5.0) was measured by LC-MS and calculated according to the standard curve, and the final concentration was 0.022 mM.

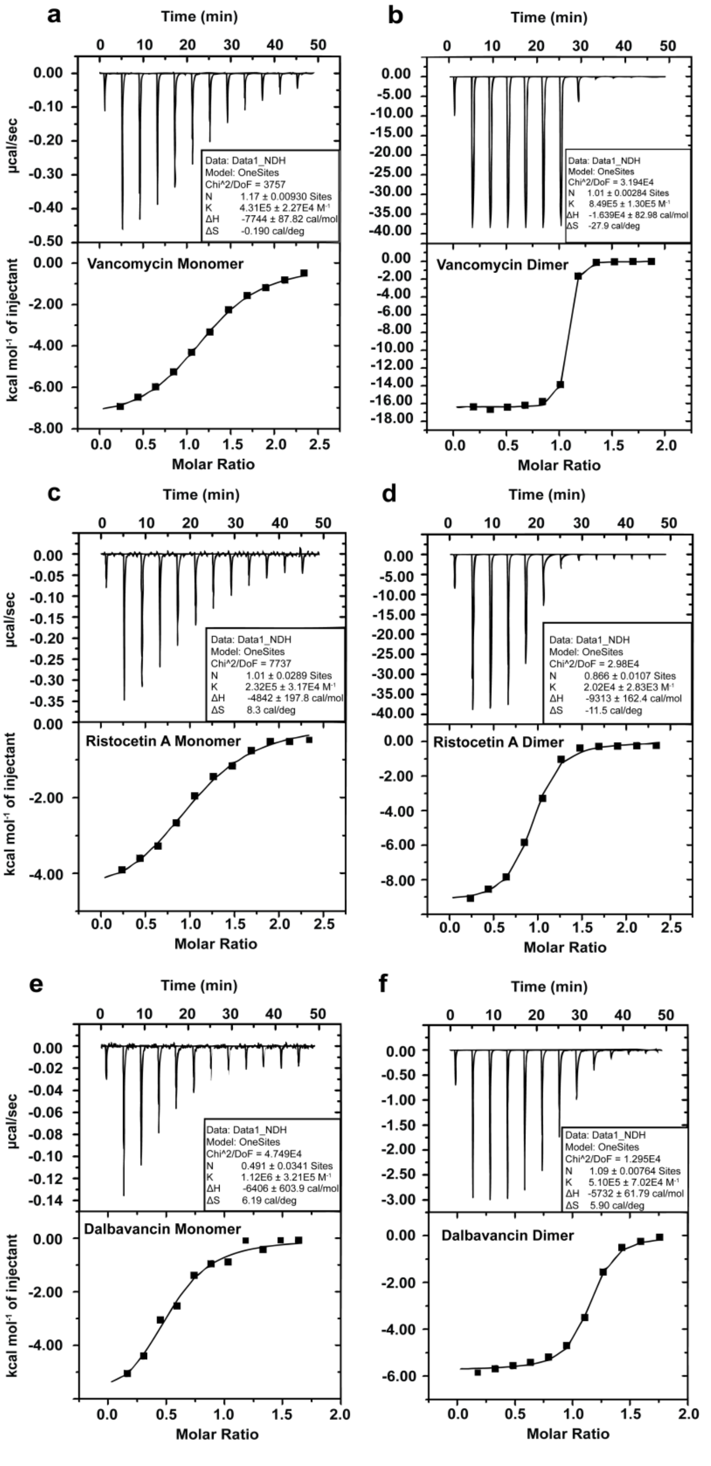

Fig. S3 Exothermic responses (upper profile) and titration curves with theoretical fit to a single site binding model as described (lower profile)[4] upon interaction of antibiotics with ligand Ac_2-Kaa in 0.1 M NaOAc (pH 5.0) at 25°C. Concentrations of vancomycin were 0.025 mM and 2 mM to assure that vancomycin exists in solution in monomeric (a) and dimeric (b) forms, respectively. Ristocetin A existed in either monomeric (c) at 0.025 mM or dimeric (d) forms at 2 mM. Concentrations of dalbavancin were 0.01 mM and 0.2 mM to allow dalbavancin to exist in monomeric (e) and dimeric (f) forms, respectively. The stoichiometry (N), association constant (K), enthalpy (ΔH) and entropy (ΔS) are derived from computer simulations.

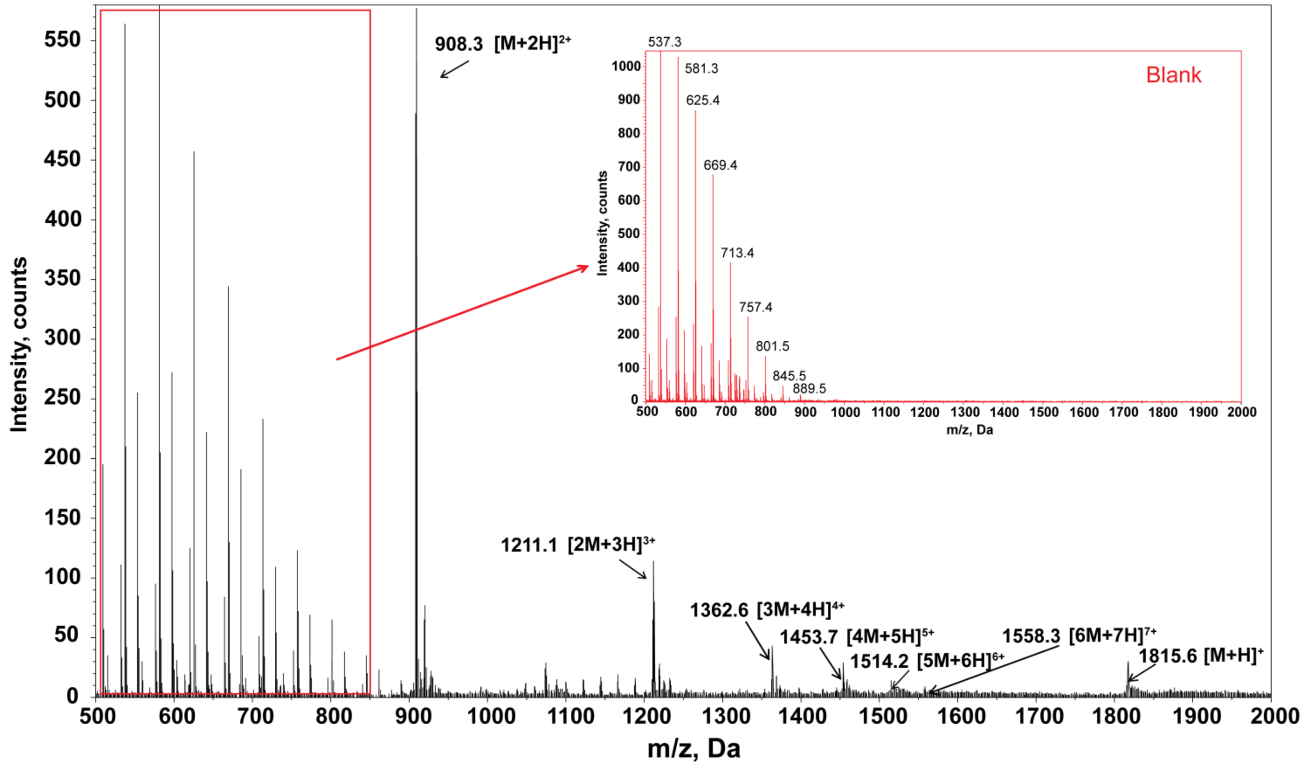

Fig. S4 ESI-MS spectrum of dalbavancin (1 mM) in H_2O-isopropanol (v/v = 8/2). The multimers of antibiotic are present as $[nM + (n+1)H^{(n+1)+}]$ mass ion species. The impurities (*m/z* ranging from 500 to 800 Da) are from the eluent (highlighted in red).

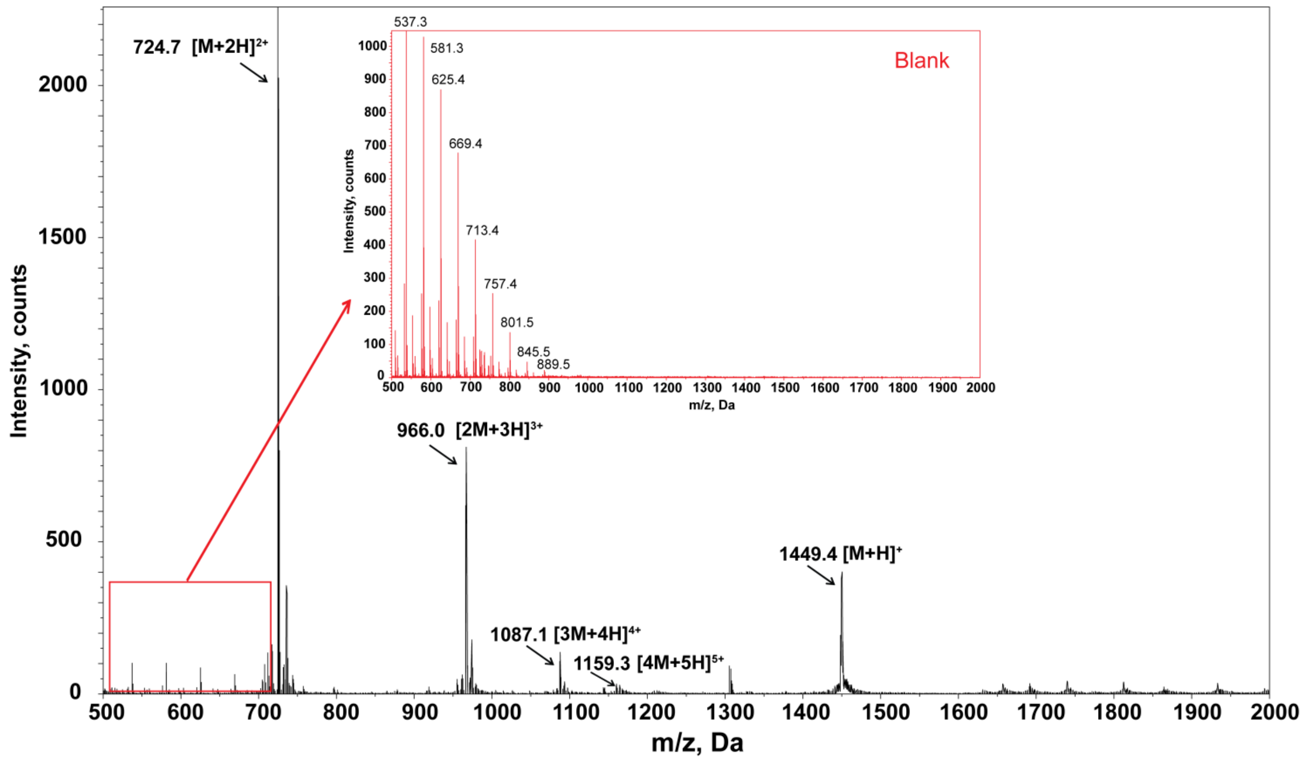

Fig. S5 ESI-MS spectrum of vancomycin (10 mM) in H_2O-isopropanol (v/v = 8/2). The impurities (*m/z* ranging from 500 to 800 Da) are from the eluent (highlighted in red).

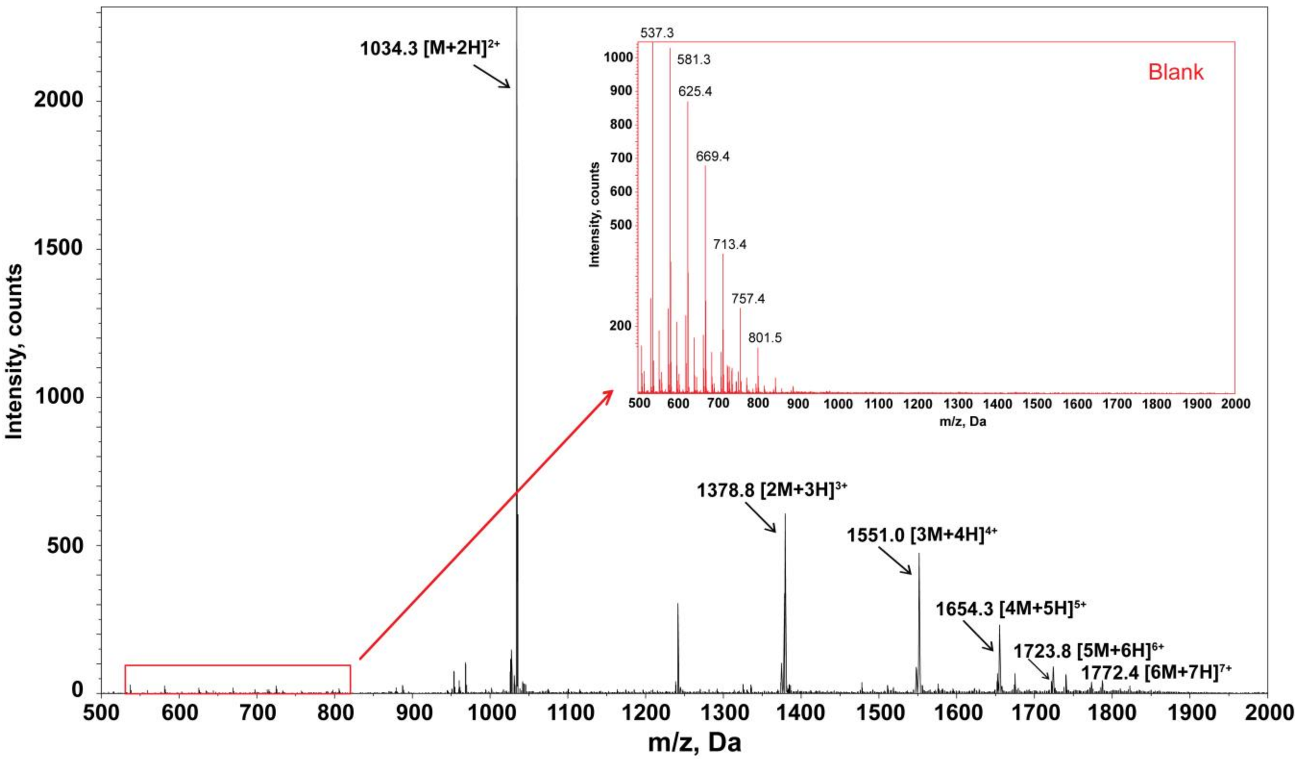

Fig. S6 ESI-MS spectrum of ristocetin A (10 mM) in H_2O/isopropanol (v/v = 8/2). The impurities (*m/z* ranging from 500 to 800 Da) are from the eluent (highlighted in red).

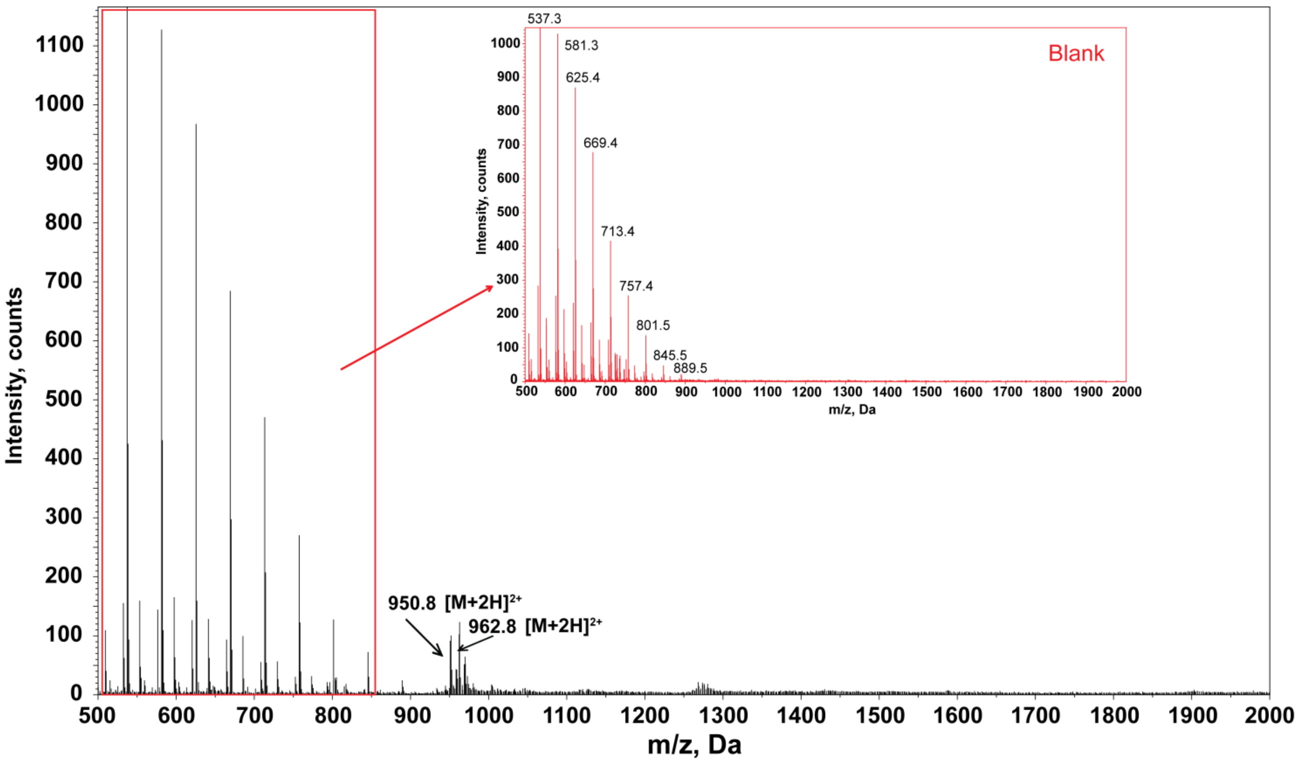

Fig. S7 ESI-MS of teicoplanin complex (A_2 major component) (10 mM) in H_2O-isopropanol (v/v = 8/2). The impurities (*m/z* ranging from 500 to 800 Da) are from the eluent (highlighted in red).

Supplementary Information References

1. A. K. Ghose, V. N. Viswanadhan and J. J. Wendoloski, *J. Phys. Chem. A*, 1998, **102**, 3762–3772.
2. National Committee for Clinical Laboratory Standards, *National Committee for Clinical Laboratory Standards, Wayne, Pa, 7th ed., M7-A7*, 2006.
3. G. G. Zhanel, D. Calic and F. Schweizer, *Drugs*, 2010, **71**, 526–526.
4. M. Rekharsky, D. Hesek, M. Lee, S. O. Meroueh, Y. Inoue and S. Mobashery, *J. Am. Chem. Soc.*, 2006, **128**, 7736–7737.
